# The Effect of Remimazolam Compared to Sevoflurane on Postoperative Shivering in Patients Undergoing Laparoscopic Gynecologic Surgery under General Anesthesia: A Prospective Randomized Controlled Trial

**DOI:** 10.3390/medicina59030578

**Published:** 2023-03-15

**Authors:** Cheol Lee, Cheolhyeong Lee, Hayoung Lee, Jeongki Park, Junsung Lim, Hyungtae Kim

**Affiliations:** 1 Department of Anesthesiology and Pain Medicine, Wonkwang University School of Medicine, 895 Muwang-ro, Iksan 54538, Republic of Korea; 2 Department of Anesthesiology and Pain Medicine, Asan Medical Center, University of Ulsan College of Medicine, Seoul 05505, Republic of Korea

**Keywords:** complication, hypothermia, remimazolam, shivering

## Abstract

*Background and objectives****:*** Anesthesia maintenance agents affect the incidence of postoperative shivering (PS) after general anesthesia. This study compared the effects of remimazolam with sevoflurane on PS in patients undergoing laparoscopic gynecologic surgery under general anesthesia. *Materials and methods*: Seventy-four patients were allocated into one of two groups. In anesthesia maintenance, group S received sevoflurane and remifentanil, and group R received remimazolam and remifentanil. *Results:* The incidence and severity of postoperative shivering, mean arterial pressure (MAP), heart rate (HR), core body temperature, and the association of PS with hypothermia, MAP, or HR in the post-anesthesia care unit (PACU) were measured. Group R had significantly lower rates of perioperative hypothermia (58.8 vs. 27.8%, *p* = 0.009) and postoperative shivering (41.2 vs. 19.4%, *p* = 0.047). The severity of PS was also lower in group R than in group S (*p* = 0.034). Core body temperature was significantly higher in group R than in group S from 10 min after induction (*p* = 0.047) to the PACU (*p* = 0.009). MAP and HR were significantly higher in group R than in group S from 20 min after induction (*p* = 0.047) to the PACU (*p* = 0.009). In group S, the correlation between the severity of PS and the incidence of hypothermia (φc = 0.414, *p* = 0.121) was moderate but not significant. In group R, the correlation between PS severity and hypothermia (φc = 0.418, *p* = 0.043) was moderate and significant. *Conclusions:* Remimazolam showed better results than sevoflurane in anesthesia maintenance regarding hypothermia and PS.

## 1. Introduction

Inadvertent perioperative hypothermia and postoperative shivering (PS) are common after anesthesia, regardless of the anesthesia methods utilized [1,2,3,4]. The incidence of perioperative hypothermia and PS after general anesthesia has been reported to be 37.5–77.2% and 20–70%, respectively. Shivering begins when vasoconstriction, a significant mechanism for preventing body heat loss, is insufficient. PS is an important complication in hypothermic patients and is one of the most common discomforts in the post-anesthesia care unit (PACU) after receiving general anesthesia [3,4].

Volatile or intravenous anesthetics impair autonomic thermoregulatory vasoconstriction, which usually preserves core body temperature (BT) [5,6]. However, benzodiazepines such as midazolam, do not significantly impair thermoregulatory control, even when combined with ordinary opioid doses [2]. In a recent study, remimazolam, a novel benzodiazepine, was found to influence thermoregulatory vasoconstriction thresholds and onset times during robotic and laparoscopic radical prostatectomy procedures. It decreased the vasoconstriction threshold to less than that of propofol, an intravenous anesthetic, and the onset of vasoconstriction was faster than that of propofol [7].

We hypothesized that remimazolam had less of an effect on thermoregulatory control than sevoflurane, a volatile anesthetic—eventually lowering the incidence of hypothermia. Therefore, this study compared the impact of remimazolam and sevoflurane on the incidence and severity of PS in patients undergoing laparoscopic gynecologic surgery under general anesthesia.

## 2. Materials & Methods

### 2.1. Study Design and Setting

This prospective controlled study was reviewed and approved by the Institutional Review Board. Informed written consent was obtained from all patients, and the trial was registered at https://clinicaltrials.gov (accessed on 28 February, 2022) (NCT05523037). Seventy-four patients—aged between 19 and 65 years, with an ASA physical status classification of I or II, who underwent laparoscopic gynecologic surgery—were included in this study.

Patients with a history of thyroid disease, cardiopulmonary disease, blood coagulation disorder, liver dysfunction, cranial nerve disease, alcohol or drug abuse, a known allergy to the study drug, and those who had a core BT >38 °C or <36.5 °C, a BMI >30 kg/m^2^, or febrile illness were excluded. Patients who underwent surgery for less than 60 min or more than 120 min and those who did not consent to participate in this study were also excluded.

Randomization, with two block sizes, was stratified using 1:1 allocation (using computerized random numbers). All patients were randomly allocated to either the sevoflurane group (group S) or the remimazolam group (group R). The attending anesthesiologists who checked the outcomes in the PACU were blinded to the study protocol.

### 2.2. Anesthesia Protocol

Each patient’s core BT was measured before anesthesia induction using an infrared tympanic membrane (TM) thermometer (ThermoScan IRT 4520, Braun, Melsungen, Germany), and the highest temperature among the three measurements was considered. A thermometer was used in the patient’s right ear. After removing the earwax, the BT of the TM core was measured using an otoscope. After anesthesia induction, a nasopharyngeal temperature probe (Temperature Probe 400 series; General Electric, Helsinki, Finland) was inserted through the nostril at an appropriate location of 9.5 to 10 cm in depth. The core BT was checked every 10 min until the end of the surgery.

In both groups, anesthesia was induced by a continuous infusion of 6 mg·kg^−1^·h^−1^ of remimazolam. The anesthesia was maintained at a BIS between 40 and 60, according to acceptable hemodynamic limits (MAP ± 30% or HR ± 30%). Group S was administered an end-tidal concentration of 1 minimum alveolar concentration (MAC) of sevoflurane, and the concentration was adjusted by 1 Vol% using a stepwise titration method. In group R, 1–2 mg·kg^−1^·h^−1^ of remimazolam was infused continuously. In both groups, all patients were administered an effect-site concentration of 3 ng·ml^−1^ of remifentanil until the end of surgery. The infusion of Remifentanil began with a target-controlled infusion system (Orchestra^®^ from Fresenius Vial, Brezinis, France), which is based on Minto’s conceptual model. After confirming that the BIS was <60, 0.9 mg·kg^−1^ of rocuronium was administered intravenously to facilitate orotracheal intubation. Mechanical ventilation was performed with a tidal volume of 8 mL·kg^−1^ and a frequency of 12 breaths.min^−1^. While the maintenance of anesthesia, heat, and humidification was performed using a heat and moisture exchanger, and the fresh gas flow rate was maintained at 2 L·min^−1^.

The ambient temperature of the operating room was maintained at 20–22 °C, and the corresponding relative humidity was 50–60%. Warmed intravenous and irrigating fluids and cold, dried peritoneal insufflation gas (CO_2_), with a constant gas flow of 200–400 mL·min^−1^, were administered during surgery. Both groups received lactated Ringer’s solution at 10 mL/kg/h for fluid deficits. A cotton blanket was applied to all the patients to promote heat retention. Intraoperative hypothermia was defined as a core BT of less than 36 °C in patients undergoing surgery under general anesthesia.

A MAP of less than 60 mmHg was considered hypotension, and an HR of <50 beats/min was considered bradycardia. A total of 250 mL of lactated Ringer’s solution was administered to treat hypotension, and, if hypotension was not corrected, 10 mg of ephedrine was administered. Atropine (0.5 mg) was administered for the treatment of bradycardia. Patients treated with ephedrine or atropine were excluded from this study.

Each patient was administered analgesics using a PCA pump containing morphine (60 mg), ketorolac (150 mg), and ramosetron (0.6 mg) at a total volume of 100 mL of saline. This device was set to deliver a basal infusion of 2 mL·hr^−1^ and bolus doses of 0.5 mL with a 15 min lockout period. An intravenous dose of ketorolac (30 mg) was administered if patients reported a VAS score ≥40, and 15 mg ketorolac was added as needed during the patients’ PACU stay.

As soon as the patients arrived at the PACU, their core BT was measured thrice using an infrared tympanic thermometer; the highest value was used as the core BT. An attending anesthesiologist observed shivering every 5 min until 15 min after the patient arrived at the PACU and every 10 min until the patient was discharged. A forced-air warming blanket (Bair Hugger Blanket, Augustine Medical, Inc., Eden Prairie, MN, USA) was used to deliver forced air at 42 °C when the core BT was below 36 °C. By resetting the warming blanket to 38 °C when the core BT reached 36 °C or higher, forced air was administered to the patient.

The incidence and severity of PS were assessed during patients’ stay in the PACU. The seriousness of PS was assessed using a bedside shivering assessment scale, which rated shivering as none (grade 0), no shivering noted on palpation of the masseter, neck, or chest wall; mild (grade 1), shivering localised to the neck and thorax only; moderate (grade 2), shivering involved the gross movement of the upper extremities (in addition to neck and thorax); and severe (grade 3), shivering involved gross movements of the trunk and upper and lower extremities [8]. If patients had a PS grade of 2 or higher, 25 mg of meperidine was administered intravenously.

### 2.3. Outcome Measurement

The primary outcome was PS incidence in the PACU. Secondary outcomes included the severity of PS in the PACU, MAP, HR, and core BT from pre-induction to the end of the surgery; the incidence of hypothermia; the association of PS with hypothermia; and meperidine administered for the treatment of PS in the PACU.

### 2.4. Statistical Analysis

The sample size was calculated using the PASS 2008 software (NCSS, Kaysville, UT, USA). A preliminary investigation showed that the incidence rates of PS in the PACU, as a primary outcome in groups S and R, were 50% and 14.3%, respectively. The results indicated that a sample size of 33 patients for each group would allow for the detection of a significant difference with a power of 90% and an α-coefficient of 0.05. Considering the dropout rate of 10%, the final sample size for each group was 37 patients. SPSS (version 25.0; SPSS Inc., Chicago, IL, USA) was used for statistical analysis. Data are presented as mean (M) ± standard deviation (SD), estimated mean (M) ± standard error (SE), or number and percentage (%). For continuous variables, the groups were compared using Student’s *t*-test or the Mann–Whitney *U* test, according to the Kolmogorov–Smirnov test of the normality of the distribution. Categorical variables were analyzed using the chi-squared test. Repeated measures ANOVA was used to analyze changes in mean scores over three or more time points. According to Mauchly’s test of sphericity, multivariate tests or tests of within-subject effects were performed. Associations between two categorical variables were analyzed using phi (φ) or Cramer’s V (φ_c_). Point-biserial correlation (*r_pb_*) was used to analyze associations between one continuous variable and one dichotomous variable.

## 3. Results

### 3.1. Study Population

**Figure 1:**
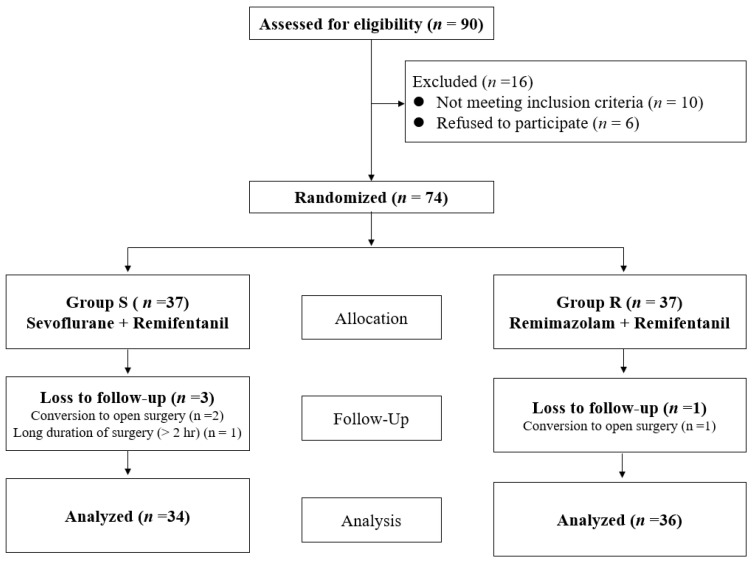
CONSORT flow diagram.

Ninety patients were assessed for eligibility, and 16 patients were excluded because they did not meet the inclusion criteria or refused to participate. Seventy-four patients were randomly assigned to each group, and four patients were lost to follow-up due to conversion to open surgery or a long duration of surgery. Finally, 70 patients were included in the analyses, as shown in the CONSORT flow diagram (Figure 1).

No significant differences in age, ASA physical status, total fluid administered, duration of anesthesia, duration of surgery, hypotension, or bradycardia were observed between the two groups. The incidence of perioperative hypothermia (*p* = 0.009) and PS (*p* = 0.047) were significantly lower in group R than in group S. The severity of PS (*p* = 0.034) and the frequency of meperidine administration to treat PS (*p* = 0.004) were also lower in group R than in group S (Table 1 and Table 2).

### 3.2. Primary and Secondary Outcomes

**Figure 2:**
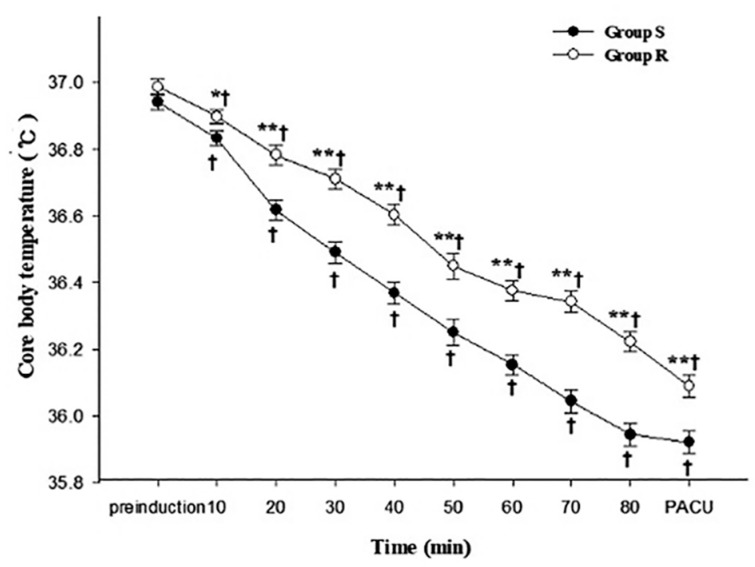
Core body temperature changes were measured in the nasopharynx (during surgery) and tympanic membrane (pre-induction and PACU). PACU, post-anesthesia care unit. Data are presented as estimated mean ± SE. * *p* < 0.05 vs. group S, ** *p <* 0.01 vs. group S, ^†^ *p* < 0.01 vs. pre-induction.

Core BT was significantly higher in group R than in group S from 10 min after induction (*p* = 0.047) to PACU (*p* = 0.009). In both groups, the core BT decreased significantly over time (*p* = 0.009) (Figure 2).

**Figure 3:**
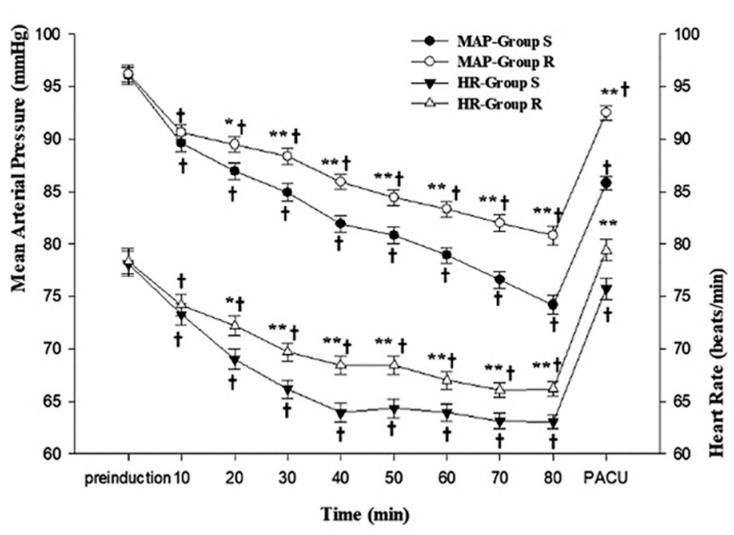
Mean arterial pressure and heart rate changes. PACU, post-anesthesia care unit. Data are presented as estimated mean ± SE. * *p* < 0.05 vs. group S, ** *p <* 0.01 vs. group S, ^†^ *p <* 0.01 vs. pre-induction.

Patients’ MAP (*p* = 0.024) and HR (*p* = 0.025) were significantly higher in group R than in group S from 20 min after induction to the PACU (*p* = 0.025, *p* = 0.013). In both groups, the MAP and HR during surgery decreased significantly over time (*p* < 0.001). In group R, patients’ HRs in the PACU returned to pre-induction levels (Figure 3).

In the PACU, the incidence of PS was significantly and moderately associated with MAP (*r_pb_* = 0.291, *p* = 0.014), HR (*r_pb_* = 0.331, *p* = 0.005), and hypothermia (φ = 0.378, *p* = 0.001) (data not shown). Patients with PS showed a higher HR than those without PS (*p* = 0.005). The association between the severity of PS and the incidence of hypothermia (φ_c_ = 0.401, *p* = 0.010) was moderate and significant. In group S, the correlation between PS severity and the incidence of hypothermia (φ_c_ = 0.414, *p* = 0.121) was moderate but not significant. In group R, the correlation between PS severity and hypothermia (φ_c_ = 0.418, *p* = 0.043) was moderate and significant (Table 3).

The association between the severity of PS requiring meperidine treatment and the incidence of hypothermia (φ_c_ = 0.329, *p* = 0.006) was moderate and significant. In group S, the correlation between PS severity requiring meperidine treatment and the incidence of hypothermia (φ_c_ = 0.195, *p* = 0.255) was moderate but not significant. In group R, the correlation between PS severity requiring meperidine treatment and hypothermia (φ_c_ = 0.391, *p* = 0.019) was moderate and significant (Table 4).

## 4. Discussion

This study demonstrated that group R showed lower rates of incidence and severity of PS, the incidence of hypothermia, and the frequency of meperidine administered for PS treatment compared to group S. The incidence of PS or PS severity requiring meperidine treatment was moderately associated with the incidence of hypothermia in group R but not in group S. Maintaining anesthesia with remimazolam compared to sevoflurane showed better results for hypothermia and PS after general anesthesia, showing a correlation between hypothermia and PS. These results may be due to the higher vasoconstriction threshold and faster vasoconstriction time of remimazolam, a benzodiazepine, compared to other inhalational or intravenous anesthetics [7].

When it comes to intravenous drugs, the reduction in thermoregulatory thresholds for shivering and vasoconstriction usually occurs in a linear dose-dependent manner. However, with volatile anesthetics, the thresholds for vasoconstriction and shivering tend to decrease in a non-linear fashion as the concentration of the volatile gas increases [2]. A previous study [5] showed that volatiles non-linearly impair thermoregulation, with an exponential increase in impairment at concentrations above 0.5 MAC. Impaired thermoregulation may differ from other forms of anesthetic dose management, such as high opioids and low volatile anesthetic doses (less than 0.5 MAC).

The clinical impact of PS includes obvious discomfort and postoperative complications, such as pain, bleeding, critical ischemia, infection, delayed wound healing, and increased hospital stays [3,4,9,10]. Both thermoregulatory and non-thermoregulatory mechanisms cause PS [4,10,11,12]. Thermoregulatory PS is usually inversely correlated with core BT and is induced by hypothermia. Approximately 15% of PS is non-thermoregulatory; is associated with decreased sympathetic nervous activity, pain, or administration of anesthetic drugs (sevoflurane or desflurane); and may occur in some normothermic patients. In the present study, remimazolam showed a moderate correlation between hypothermia and PS compared with sevoflurane, suggesting that the thermoregulatory mechanism contributed more than the non-thermoregulatory mechanism.

Our previous study [7] found that remimazolam had a higher vasoconstriction threshold and a faster onset of vasoconstriction than propofol. However, intraoperative hypothermia was not different when the operation lasted longer than 3 h. The duration of the operation is also an important factor affecting hypothermia during surgery [1]. The results of the present study showed that remimazolam showed better outcomes in 1 to 2 h surgeries, in terms of intraoperative hypothermia and PS, than sevoflurane.

Kiekkas et al. [10] reported the possible impacts of hypothermia and shivering on MAP and HR. Volatile anesthetics (sevoflurane) and intravenous analgesics (remifentanil) continuously administered during anesthesia maintain the HR and MAP within narrow limits, according to each patient’s baseline values measured before induction of anesthesia, and these control potentials are lost when the patient wakes up from anesthesia. In the present study, group R showed higher core BT, MAP, and HR during surgery than group S. As shown in previous studies [7,13], remimazolam, a benzodiazepine, shows better thermoregulatory control and hemodynamics than sevoflurane. The incidence of PS was moderately associated with MAP and HR. Patients with PS showed a higher HR than those without PS. The results of the present study are consistent with those of a previous study [10].

The present study has some limitations. First, core BT was measured using an infrared pre-induction thermometer in the PACU and a nasopharyngeal probe during surgery. However, measurement errors may occur between these two methods and sites [14]. Measurement errors may have affected the incidence of perioperative hypothermia and the association between PS and hypothermia. Second, we needed to determine whether the mucosa of the nasopharynx was the optimal location for probe placement. A misplaced nasopharyngeal temperature probe produces core BT values that differ significantly from those obtained using an optimally positioned probe [15,16]. Third, in a previous study [17], female hormonal status, according to the menstrual cycle, had a weak positive correlation with hypothermia. We should have checked the menstrual cycle or female reproductive hormone levels, which could affect thermal perception and autonomic thermoregulatory responses. In addition, since sex is also one of the factors that affect thermoregulation [18], further research is needed in male patients. Finally, the net effect of sevoflurane or remimazolam could not be confirmed because of the administration of remifentanil, which is associated with an increased incidence of PS. This is probably because the vasodilatory effect of remifentanil affects thermoregulation, in a dose-dependent manner, as an underlying mechanism [4]. The effect of inhalational anesthetics used for general anesthesia on the core body temperature is not known to be affected by intravenous anesthetics used for inducing anesthesia or by intravenous anesthetics used for inducing general anesthesia. As the effect of remimazolam, which is used for anesthesia induction, on sevoflurane, which is used for anesthesia maintenance, cannot be ignored, it is imperative that more controlled studies are conducted.

## 5. Conclusions

The maintenance of anesthesia with remimazolam compared to sevoflurane showed better results regarding the incidence and severity of PS in the PACU. Remimazolam also showed a better association with hypothermia and PS than sevoflurane. Consequently, remimazolam is usually associated with thermoregulated PS, while sevoflurane may be associated with non-thermoregulatory PS, which can also be found in normothermic patients.

## Figures and Tables

**Table 1:** Patients’ demographic and perioperative data.

	Group S (*n* = 34)	Group R (*n* = 36)	*p*-Value	SMD
Age (yr)	49.0 ± 8.1	49.7 ± 6.4	0.703	−0.67
ASA (I/II)	15/19 (44.1/55.9)	20/16 (55.4/44.4)	0.339	
Body mass index (kg/m^2^)	24.0 ± 0.6	23.8 ± 0.9	0.705	0.14
Total fluid administered (mL)	698.5 ± 108.4	730.6 ± 98.0	0.199	−32.0
Duration of anesthesia (min)	95.0 ± 8.3	96.9 ± 8.7	0.398	−1.94
Type of surgery			0.94	
Laparoscopic subtotal hysterectomy	12 (35.3)	13 (36.1)		
Laparoscopic-assisted vaginal hysterectomy	22 (64.7)	23 (63.9)		
Duration of surgery (min)	76.2 ± 9.2	76.9 ± 9.6	0.7485	−0.77
Hypotension	2 (5.9)	0 (0)	0.14	
Bradycardia	0 (0)	0 (0)		

Data are presented as mean ± standard deviation (SD) or number and percentage (%). SMD, standardized mean difference. ASA, American Society of Anesthesiologists physical status. A MAP of less than 60 mmHg was considered hypotension, and an HR of <50 beats/min was considered bradycardia.

**Table 2:** The incidence of hypothermia and the severity and treatment of postoperative shivering.

	Group S (*n* = 34)	Group R (*n* = 36)	*p*-Value
Incidence of hypothermia (<36 °C)	20 (58.8)	10 (27.8)	0.009
Incidence of PS	14 (41.2)	7 (19.4)	0.047
Severity of PS			0.034
Grade 0	20 (20.8)	29 (46.7)	
Grade 1	3 (41.7)	5 (40.0)	
Grade 2	8 (33.3)	2 (13.3)	
Grade 3	3 (8.8)	0 (0)	
Meperidine administered for the treatment of PS	11 (32.4)	2 (5.6)	0.004

Data are presented as mean ± standard deviation (SD) or number and percentage (%). PS, postoperative shivering. Grade 0, no shivering noted on palpation of the masseter, neck, or chest wall; grade 1, shivering localized to the neck and thorax only; grade 2, shivering involved the gross movement of the upper extremities (in addition to the neck and thorax); and grade 3, shivering involved gross movements of the trunk and upper and lower extremities.

**Table 3:** The association between the severity of PS and the incidence of hypothermia in the PACU.

		No Hypothermia	Hypothermia	Cramer’s V (φ_c_) Coefficient	*p*-Value
Group S	PS			0.414	0.121
	Grade 0	11 (78.6)	9 (45.0)		
	Grade 1	0 (0.0)	3 (15.0)		
	Grade 2	3 (21.4)	5 (25.0)		
	Grade 3	0 (0.0)	3 (15.0)		
Group R	PS			0.418	0.043
	Grade 0	23 (88.5)	6 (60.0)		
	Grade 1	3 (11.5)	2 (20.0)		
	Grade 2	0 (0)	2 (20.0)		
Total	PS			0.401	0.010
	Grade 0	34 (85.0)	15 (50.0)		
	Grade 1	3 (7.5)	5 (16.7)		
	Grade 2	3 (7.5)	7 (23.3)		
	Grade 3	0 (0)	3 (10.0)		

Data are presented as number and percentage (%). PS, postoperative shivering. Grade 0, no shivering noted on palpation of the masseter, neck, or chest wall; grade 1, shivering localized to the neck and thorax only; grade 2, shivering involved the gross movement of the upper extremities (in addition to the neck and thorax); and grade 3, shivering involved gross movements of the trunk and upper and lower extremities.

**Table 4:** The association between the severity of PS requiring treatment with meperidine and the incidence of hypothermia.

		No Hypothermia	Hypothermia	Cramer’s V (φ_c_) Coefficient	*p*-Value
Group S	PS			0.195	0.255
	≤Grade 1	11 (78.6)	12 (60.0)		
	≥Grade 2	3 (21.4)	8 (40.0)		
Group R	PS			0.391	0.019
	≤Grade 1	26 (100)	8 (80.0)		
	≥Grade 2	0	2 (20.0)		
Total	PS			0.329	0.006
	≤Grade 1	37	20 (66.7)		
	≥Grade 2	3	10 (33.3)		

Data are presented as number and percentage (%). PS, postoperative shivering. Grade 0, no shivering noted on palpation of the masseter, neck, or chest wall; grade 1, shivering localized to the neck and thorax only; grade 2, shivering involved the gross movement of the upper extremities (in addition to the neck and thorax); and grade 3, shivering involved gross movements of the trunk and upper and lower extremities.

## Data Availability

Data will be provided upon reasonable request due to privacy and ethical restrictions policy.
